# Association between occupational exposure and the clinical characteristics of COPD

**DOI:** 10.1186/1471-2458-12-302

**Published:** 2012-04-26

**Authors:** Denis Caillaud, Franck Lemoigne, Philippe Carré, Roger Escamilla, Pascal Chanez, Pierre-Régis Burgel, Isabelle Court-Fortune, Gilles Jebrak, Christophe Pinet, Thierry Perez, Graziella Brinchault, Jean-Louis Paillasseur, Nicolas Roche

**Affiliations:** 1 Service de Pneumologie, Hôpital Gabriel Montpied, Montalembert Str, Clermont-Ferrand 63003, France; 2 Service de Pneumologie, CHU de Nice, 30 voie romaine Av, Nice 06000, France; 3 Service de Pneumologie, Hôpital Antoine Gayraud, BP 1014, Carcassonne 11890, France; 4 Clinique des voies respiratoires, Hopital Larrey, 24, Pouvourville Str, Toulouse 31059, France; 5 Département des Maladies Respiratoires, AP-HM, Université de la Méditerranée, Bourelly Str, Marseille 13915, France; 6 Service de Pneumologie, Hôpital Cochin, AP-HP and Paris-Descartes University, 27, Faubourg St-Jacques Str, Paris 75014, France; 7 Service de Pneumologie, CHU Saint Etienne, Albert Raimond Av, Saint Etienne 42055, France; 8 Service de Pneumologie, Hôpital Bichat, 46, Henri Huchard Str, Paris 75018, France; 9 Service de Pneumologie, CHG, 1208, colonel Picot Av, Toulon 83056, France; 10 Service de Pneumologie, Hôpital Calmette, J Leclerq bd, Lille 59037, France; 11 Service de Pneumologie, Hôpital Pontchaillou, 2, Henri Le Guillou Str, Rennes 35000, France; 12 Clindatafirst, 12, Prés Av, St Quentin en Yvelines 78059, France; 13 Service de Pneumologie, Hôtel Dieu, AP-HP and Paris-Descartes University, Parvis Notre Dame Place, Paris 75004, France

**Keywords:** Occupational, COPD, Exposure assessment

## Abstract

**Background:**

The contribution of occupational exposures to COPD and their interaction with cigarette smoking on clinical pattern of COPD remain underappreciated. The aim of this study was to explore the contribution of occupational exposures on clinical pattern of COPD.

**Methods:**

Cross-sectional data from a multicenter tertiary care cohort of 591 smokers or ex-smokers with COPD (median FEV1 49%) were analyzed. Self-reported exposure to vapor, dust, gas or fumes (VDGF) at any time during the entire career was recorded.

**Results:**

VDGF exposure was reported in 209 (35%) subjects aged 31 to 88 years. Several features were significantly associated with VDGF exposure: age (median 68 versus 64 years, p < 0.001), male gender (90% vs 76%; p < 0.0001), reported work-related respiratory disability (86% vs 7%, p < 0.001), current wheezing (71% vs 61%, p = 0.03) and hay fever (15.5% vs 8.5%, p < 0.01). In contrast, current and cumulative smoking was less (p = 0.01) despite similar severity of airflow obstruction.

**Conclusion:**

In this patient series of COPD patients, subjects exposed to VDGF were older male patients who reported more work-related respiratory disability, more asthma-like symptoms and atopy, suggesting that, even in smokers or ex-smokers with COPD, occupational exposures are associated with distinct patients characteristics.

## Background

Chronic obstructive pulmonary disease (COPD) is currently viewed as a consequence of interactions between genetic and environmental factors. Tobacco smoking is the most important risk factor for COPD [1], but other environmental factors such as occupational exposures are likely to contribute in some patients [2]. In 2010, the American Thoracic Society estimated that more than 20% of COPD cases are attributable to occupational exposure [3].

Most studies documenting the association between COPD and occupational exposures have been performed in workplace settings or in general population samples and community-based cohorts. Only one patient series specifically assessed the characteristics of COPD patients with a history of occupational exposures. Historically, the identification of work-related COPD has been based on the observation of an excess occurrence of COPD among exposed workers in specific industries [4]. Subsequently, general population-based studies such as the ECRHS [5] or community-based cohorts of patients with COPD [6-8] found an increased risk of COPD in subjects exposed to vapors, dust, gas, fumes (VDGF) or working in agricultural, paper, cleaning, wood and food processing industries. In the only available series of COPD patients (n = 185), VDGF exposure was associated with sputum production and increased dyspnea [9]. In most studies, occupational exposures were assessed using questionnaires with broad terms encompassing multiple exposures (VDGF), which appear reasonably effective in identifying subjects significantly exposed to occupational air pollutants [10]**.**

Regarding the clinical and lung function characteristics of COPD, it might be hypothesized that occupational exposures could be associated with specific phenotypic traits such as atopy, since many occupational air pollutants have sensitizing properties that could interact with genetic predisposition [11]. However, this hypothesis has not been adequately tested yet.

The present analysis was designed to explore the influence of occupational exposures on disease characteristics in in a patient series of 591 smokers or ex-smokers with well-defined COPD.

## Methods

### Population

The present study is based on the analysis of cross-sectional data from a previously described cohort of COPD patients recruited between January 2005 and August 2008 in 17 university respiratory medicine departments located throughout France [12]. Respiratory physicians prospectively recruited smokers or ex-smokers (>10 pack-years cumulative tobacco consumption) in stable condition (no history of exacerbation requiring medical treatment for the previous 4 weeks) with a GOLD (Global Initiative on Obstructive Lung Disease)-based diagnosis of COPD (post-bronchodilator FEV_1_/FVC ratio < 70%) [13]. Patients with a main diagnosis of bronchiectasis, asthma or any significant respiratory disease other than COPD were excluded. The study was approved by the Ethics Committee of Versailles (France) and all patients provided informed written consent.

### Data collection

We used a standardized case report form that covered demographic data, cumulative tobacco smoking, and clinical and lung function characteristics in stable condition. Symptoms of chronic bronchitis and dyspnea were evaluated using questions derived from the European Community Respiratory Health Survey (ECRHS). Current (during the previous year) history of sputum production and wheezing was assessed [14]. Pulmonary function tests were performed according to international standards [15]. The number of self-reported acute exacerbations of COPD was assessed and exacerbations were classified as mild, moderate (use of antibiotics or oral corticosteroids) or severe (hospitalization or emergency department visit). Health related quality of life was evaluated using the Saint George’s Respiratory Questionnaire (SGRQ) [14].

### Risk factors

Prior occupational exposures were assessed by the following ECRHS-derived question: “Have you ever worked in a job which exposed you to vapors, dust, gas, or fumes?” [5]. A history of transient work-related respiratory disability was assessed by the question: “Have you ever had to stop transiently your job because it affected your breathing? “Permanent work-related respiratory handicap was defined by a positive answer to the question “Have you ever had to stop definitely your job because it affected your breathing?”. Patients who reported VDGF exposure were questioned on the job position that they occupied during the longest period of time. Answers were analyzed using a previously published specific job exposure matrix (JEM) that provided a semiquantitative estimate (none, low or high) of exposure to three broad categories of agents: organic dust, mineral dust, or gas/fumes [16,17].

Patients were categorized (i) as current smokers or ex-smokers (smoking cessation at least 12 months ago) and (ii) as smokers or ex-smokers of ≥ or < 20 pack-years [16].

Patients were considered as atopic when they reported a history of hay fever.

### Statistical analysis

Since some variables were not continuously distributed, data are reported as median [Q1-Q3] for quantitative variables. Percentages were used to describe categorical variables. Univariate comparisons between patients exposed or not to VDGF were performed using Chi-square test for qualitative variables and the two-sided non-parametric Wilcoxon test for quantitative variables.

A p value <0.05 was considered for statistical significance. Analysis was performed with SAS software (version 9.2).

### Ethic statement

The research protocol was approved by the ethics committee of Versailles (France) and all patients provided informed written consent.

## Results

### Analysis of demographic characteristics and risk factors in COPD subjects according to VDGF exposure

Among the 615 COPD patients included in the cohort, required data were available in 591 (96%). Exposure to VDGF was reported by 209 subjects (35%) and was more common among males than females (90% vs 10%, Table 1). The median age was 65 years, patients reporting VDGF exposure being about 4 years older than VDGF- subjects. Altogether, 27% of patients were still current smokers at inclusion in the cohort. Cumulative smoking was 41 (26–57) pack-years. Current and cumulative smoking were also less in COPD patients with VDGF exposure, despite similar severity of airflow obstruction (see following table). Cumulative smoking and FEV1 (% predicted) were not correlated (r = 0.08). With regard to atopy, hay fever was almost twice more frequent in the VDGF + population. A trend towards a similar association was found for life-time asthma. FEV1 and GOLD stage did not differ between exposed and non-exposed subjects. They were not influenced by the level of cumulative smoking.

**Table 1:** Characteristics of the study population (N = 591): data are median [Q1-Q3]

	**All n = 591**	**VDGF + n = 209 (35%)**	**VDGF - n = 382 (65%)**	**P**
Male/female ratio (%)	80.7/19.3	89.9/9.1	75.6/24.4	**<0.001** (a)
Age	65 [57–73]	68 [59–74]	64 [57–72]	**0.0005** (b)
Smoking status Current/past (%)	27/73	22/78	29/71	**0.04** (a)
Smoking, pack-yr	41 [26–58]	39 [20–54]	42 [28–60]	**0.01** (b)
Hay fever	11	15.5	8.5	**<0.01** (a)
Life-time atopic dermatitis	7	8 .8	6.1	NS (a)
Life-time asthma	11.3	14.7	9.5	0.0614 (a)

(a) = Chi-Square Test.

(b) = Wilcoxon rank-sum test.

p values are for differences between VGDF + and VGDF-.

Among patients reporting VDGF exposure, job-exposure matrix (JEM) analysis found that 61% were exposed to mineral dusts, 34% to biological pollutants and 73% to gas, vapors or fumes. Altogether, 3% of the whole patient population were exposed to one category of pollutants, 20% to two and 8% to three categories. Current smokers were less represented among subjects exposed to mineral dusts (17% vs 30% in the remaining population) and to vapors, gas and fumes (20% vs 29%).

### Symptoms and health status

In terms of symptoms, although chronic bronchitis was not more frequent among VDGF + COPD patients (overall prevalence: 73%), they reported current sputum production more often. Similarly, current wheeze and life-time wheeze were more frequent in patients with VDGF exposure, which was associated with poorer health status (Table 2).

**Table 2:** Symptoms according to VDGF exposure

	**All n = 591**	**VDGF + N = 209**	**VDGF – 382**	**P value**
Chronic bronchitis (%)	73.3	75.3	72.2	NS (a)
Shortness of breath (MRC)	2 [1–2]	2 [1–2]	2 [1–2]	NS (b)
Life-time wheeze (%)	65	71.9	61.1	**0.0371 (a)**
Exacerbations/patient/yr	1 [0–3]	1 [0–3]	1 [0–3]	NS (b)
FEV_1_ (postBD, % predicted)	49 [35–66]	51.6 [37.2–67.1]	47.2 [33.7–65.3]	0.08 (b)
GOLD stages (%)				0.15 (a)
I	5.6	7.7	4.5	
II	43	44.5	42.2	
III	33.2	33.5	33	
IV	18.3	14.4	20.4	
Current sputum^1^	2 [1–4]	3 [1 – 4].	2 [1 – 4].	**0.035 (b)**
Current wheeze^1^*	1 [0–2]	2 [0 – 3].	1 [0 – 2].	**0.002 (b)**
Work related respiratory disability *	1 [0–1]	1 [0 – 2].	0 [0 – 1].	**0.001 (b)**
SGRQ total score *	43.7 [30.3-60.0]	46.2 [32.5-63.4]	42.0 [29.9-58.2]	NS (b)

^1^: during the previous year (answers to SGRQ questions, symptoms domain).

*: analysis restricted to patients with available SGRQ: n = 424 (VDGF+: n = 144; VDGF-: n = 280). Answers were converted to continuous variables. Data are median [Q1 – Q3] or %.

(a) = Chi-Square Test.

(b) = Wilcoxon rank-sum test.

p values are for differences between VGDF + and VGDF.

### Work disability

COPD patients with VDGF exposure reported significantly more often an impact of their respiratory situation on their ability to work: 28.5% of VDGF-exposed subjects had to stop working definitely due to breathing difficulties vs 20.3% in non-exposed patients. In addition, 34.7% (vs 25.4%) had to stop working transiently because they experienced respiratory problems. Altogether, only 36.8% of VDGF-exposed subjects (vs 54.3% of non-exposed subjects) reported no impact of their breathing situation on work.

**Figure 1:**
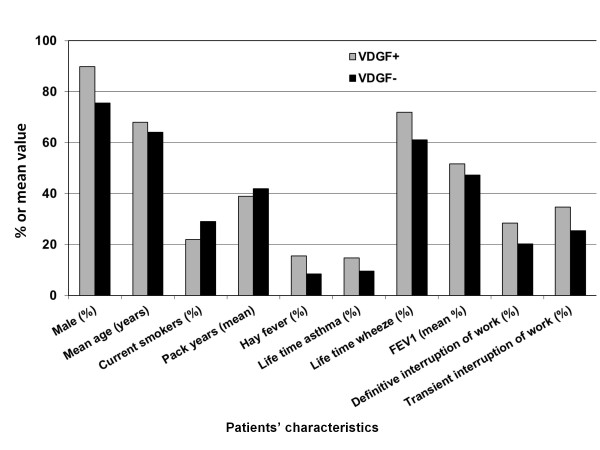
**Main differences between VDGF + and VDGF- COPD patients.** All differences are significant at the p < 0.05 level except life time asthma (p = 0.06) and FEV1(p = 0.08). Reported definitive and transient interruptions of work are those related to respiratory problems.

Main differences between VDGF + and VDGF- COPD patients are summarized in Figure 1.

## Discussion

In this patient series, the clinical characteristics of COPD appeared to be modulated by occupational exposures: patients reporting exposure to vapors, dust, gas or fumes at some point during their career also reported more frequently a history of hay fever, current wheeze and transient or permanent occupational disability. They were older and predominantly men with less current and cumulative smoking, despite similar severity of airflow obstruction. This is the first patient series identifying such specific clinical characteristics associated with occupational exposures in smokers and ex-smokers with COPD.

### Role of atopy in the development of COPD related to occupational exposures

Since the late 1960s, it has been known that, although cigarette smoking is a risk factor for COPD, only 20 to 30% of cigarette smokers develop airways disease, suggesting that genetic susceptibility plays an important role as a determinant of disease occurrence.

In our cohort, occupational exposures were associated with atopy, despite the fact that all COPD patients were smokers or ex-smokers. This observation suggests that atopy and at least some occupational exposures interact to favour the development of the disease. Some decades ago, the Dutch hypothesis suggested that asthma and COPD are different manifestations of a single disease entity, called chronic nonspecific lung disease. It was suggested that endogenous factors (e.g., sex and age), environmental factors (e.g., allergens, occupation, and smoking) and genetic factors (including those predisposing to atopy and airway hyperresponsiveness) all play a role in the pathogenesis of the disease [18,19].

Indeed, there is evidence that atopy, independently of its association with bronchial hyperesponsiveness, may be associated or have a role in the pathogenesis of COPD [18,20]. In a general population study, COPD was associated not only with smoking but also with occupational exposures and hay fever [21]. Other studies found an inverse association between atopy, as defined by IgE level, and the FEV1/FVC ratio, independently of smoking status [22]. Some genes, such as those coding IL-13 and IL-17F, might be involved in a global model of shared genetic factors for atopy, asthma and COPD [11,23]. Lastly, it could be hypothesized that, in predisposed subjects, tobacco smoking may facilitate the development of atopy through its effect on IgE levels [24]. In patients subsequently exposed to occupational sensitizing agents, this may lead to the development of asthma-like features.

### Association between occupational exposures, asthma-like symptoms and lung function

Persistent wheeze, which was associated with VDGF exposure in our series of COPD patients, can be a feature of airway hyperresponsiveness (AHR) [25]. AHR is a cardinal feature of asthma [26] and may contribute to the development of COPD [27]. Indeed, several studies found an increase in the risk of COPD in patients reporting a personal history of asthma or AHR [8,28].

A longitudinal study of males with early COPD suggested that occupational exposure to fumes could be associated with an increased rate of decline of lung function [29]. However, in our population, lung function of exposed patients did not differ from that of patients with no reported occupational exposure. This discrepancy could relate to the lower cumulative smoking in exposed patients, or to an improvement in occupational conditions with aging, related to seniority in the job [30].

### Association between occupational exposures and respiratory disability

In subjects with COPD, exposure to VDGF appears to promote transient or even permanent work loss due to respiratory disability. Only a few studies reported work loss associated with COPD. In the ECRHS, which included adults aged 20 to 44 years, job change due to breathing difficulties at work was reported by 4% of the whole studied population. This figure increased to 11% in subjects reporting either asthma or chronic bronchitis [31]. In the confronting COPD international survey (mean age 63.3 years), more than one third of persons with COPD (35.7%) reported that their condition kept them from working [32]. In a community based cohort study using structured telephone interviews of 234 COPD patients, 25% reported respiratory disability at work and 16% reported both VDGF exposures and respiratory-related work disability [6]. In a patient series of 185 male patients, 34 had become unemployed (18%) due to COPD [9]. Our patient series is in agreement with these studies, with 28.5 % of patients reporting cessation of work due to breathing, which is likely associated to both social and economical consequences.

### Accuracy of exposure assessment and other limitations and strengths of the study

Assessment of occupational exposure was relatively crude in this study, since self-reported exposure based on a single item is subject to recall bias or subjective influences, in contrast to job-exposure matrix (JEM), which is considered as the “gold standard”. However, previous studies performed in the general population suggest that, when compared to complex assessments such as job-exposure matrices, self-reported exposures are accurate to identify associations between occupation and disease [33]. For instance, in two cohort studies of adults with asthma, self-reported VDGF exposure was fairly sensitive (71%) when compared to JEM-defined exposure [34] and performed well against a checklist of 16 specific exposures [35]. In addition, several studies found increased respiratory symptoms in association with self-reported VDGF exposure [36,37]. For instance, one study of subjects with established COPD showed that prior exposure to VDGF was associated with increased symptoms over a 1 year follow-up [6]. Finally and importantly, the job-exposure matrix analysis in our subjects reporting VDGF exposure confirmed the reality of exposure. In the present study, statistical analyses using job-exposure matrix were performed to explore the relationship between the type of exposure and the presence of atopy, hay fever, asthma and wheezing; however, due to the relatively low number of patients in each individual category, it was not possible to draw any firm conclusion (data not shown).

Since patients recruited in the real-life observational Initiatives BPCO cohort are all followed in tertiary care centers, they cannot be considered as representative of the general COPD population. In addition, although centers were asked to include all consecutive COPD patients visiting their clinic, it is likely that recruitment was not exhaustive and varied with local resources affected to the study, competitive studies (subjects could not be included in the cohort if they participated to another study) and the effective duration of each center’s participation to the cohort constitution. However, although we cannot formally test the issue of selection bias, it must be noted that (i) the study protocol did not mention any specific guidance on risk factors and (ii) the proportion of patients with occupational exposures is similar to what has been reported in other cohorts. Therefore, a selection bias appears quite unlikely to influence the results of the present analyses.

Pre-bronchodilator FEV1 was not available for all subjects, which prevented us from including reversibility data in the studied variables. Patients exposed to VDGF could have more reversible airway obstruction, corresponding to the asthma-like symptoms (increased frequency of current wheeze) that were reported. However, we have no way to test this hypothesis and, in general, the relationship between symptoms and lung function variables including reversibility is poor. Along the same line, it would have been interesting to compare lung volumes, diffusing capacity, and non-specific bronchial hyperresponsiveness between exposed and unexposed patients. However, these measurements were unavailable in most subjects, as explained by the real-life nature of the cohort. Similar concerns can be expressed for skin prick tests.

A particular aspect of this patient series is that, to limit the risk of including predominantly asthmatic subjects with fixed airflow obstruction, it happened that all centers included only smokers or ex-smokers. This was both a strength since it “secured” the diagnosis of COPD, and a limitation in that it precluded analyzes of the role of occupational exposures in never-smokers. However, it did not prevent from identifying specific clinical features in exposed patients. Similar studies in never-smoking subjects with COPD would be of interest to determine whether our observations are the results of VDGF only, or of VDGF-cigarette smoke interactions.

Another area of interest is the respective weight of risk factors as determinants of the severity of airflow obstruction. Although presented analyses were not initially designed to address this research question, we explored this issue using two multivariate models: one was a logistic regression analysis with GOLD stage as dependent variable, the other was a multilinear regression with FEV_1_ (% predicted) as the dependent variable. Smoking was accounted for using cumulative consumption in pack-years and smoking status at inclusion (present vs past smoking). Variables reflecting atopy were patient-reported hay fever and familial history of atopy. With the first model, only less than 2% (R^2^ = 0,018, p value of the model: 0.46) of variations in the GOLD classification could be explained by risk factors. In the second model, R^2^ was even lower (0.0089, with a p value for the model of 0.28). Thus, it can be concluded that, in this population of smokers and ex-smokers, risk factors (age, smoking, occupational exposure, atopy) are very poorly correlated with the severity of airflow obstruction, which makes it impossible to draw any conclusion as to their respective weight.

## Conclusions

This is the first study to demonstrate that occupational exposures are associated with distinct clinical characteristics in smokers and ex-smokers with COPD, i.e. (i) older age and predominantly male gender, (ii) more frequent respiratory-related work disability or handicap and (iii) increased prevalence of hay fever, asthma, current sputum production, lifetime and current wheezing. This may help targeting subjects for preventive measures. Our findings need to be confirmed and expanded in other cohorts, which should also aim at identifying more precisely which occupational pollutants are involved and which exposure thresholds (dose, duration, frequency) are associated with an increased risk.

## Competing interest

The authors declare they have no competing interests

## Authors’ contributions

All authors participated to the study design, data collection, manuscript revision and approval of the final version. D Caillaud and N Roche wrote the paper and interpreted the data, which was analyzed by JL Paillasseur, statistician. All authors read and approved the final manuscript.

## Pre-publication history

The pre-publication history for this paper can be accessed here:

http://www.biomedcentral.com/1471-2458/12/302/prepub
